# Two Decades of Change: Evolving Maternal Characteristics and Perinatal Outcomes in Pregnant Women Living with HIV

**DOI:** 10.3390/v17111425

**Published:** 2025-10-27

**Authors:** Laura Salazar, Anna Goncé, Isabel Matas, Judit Balcells, Laura García-Otero, Clàudia Fortuny, Berta Torres, Ana González-Cordón, Montse Palacio, Eduard Gratacós, Francesc Figueras, Fatima Crispi, Marta López

**Affiliations:** 1 Department of Maternal-Fetal Medicine, Barcelona Center for Maternal-Fetal and Neonatal Medicine (Hospital Clínic and Hospital Sant Joan de Déu), 08028 Barcelona, Spainlaura.garcia@isglobal.org (L.G.-O.); fcrispi@clinic.cat (F.C.); 2 Institut d’Investigacions Biomèdiques August Pi i Sunyer (IDIBAPS), 08036 Barcelona, Spainagonzal1@clinic.cat (A.G.-C.); 3 Faculty of Medicine, Universitat de Barcelona, 08036 Barcelona, Spain; 4 Infectious Diseases Unit, Pediatrics Department, Institut de Recerca Hospital Sant Joan de Déu (Hospital Sant Joan de Déu), 08950 Esplugues de Llobregat, Spain; 5 HIV/AIDS Unit, Infectious Diseases Department, Hospital Clínic de Barcelona, 08036 Barcelona, Spain; 6 Center for Biomedical Network Research on Infectious Diseases (CIBERINFEC), Instituto de Salud Carlos III, 28029 Madrid, Spain; 7 Center for Biomedical Network Research on Rare Diseases (CIBER-ER), Instituto de Salud Carlos III, 28029 Madrid, Spain

**Keywords:** HIV, pregnancy, antiretroviral therapy, perinatal outcomes

## Abstract

Implementation of universal antiretroviral treatment (ART) in pregnancy has improved maternal health and reduced vertical transmission. However, women living with HIV (WLHIV) still experience worse perinatal outcomes. This retrospective study compared demographic, virological factors, ART regimens and perinatal outcomes in pregnant WLHIV between 2000–2010 (n = 318) and 2011–2021 (n = 140) at a tertiary center in Barcelona. Significant demographic shifts included changes in ethnic distribution, substance use, educational attainment, and maternal BMI. Significant progress in infection control was observed, with increased ART coverage up to 97%, improved viral suppression (80% to 91.3%, *p* = 0.002), and enhanced immunological status. ART regimens shifted significantly, with an increase in integrase strand transfer inhibitors (INSTI)-based regimens (0.7% to 39.2%, *p* < 0.001). Obstetric management evolved, with a rise in vaginal deliveries (24.8% to 44.3%, *p* < 0.001) and a decline in intrapartum zidovudine (93.7% to 54.7%, *p* < 0.001). Notably, preterm birth rates sharply declined, yet small-for-gestational-age (SGA) infants (26.4% vs. 20%, *p* = 0.323) and preeclampsia rates remained unchanged and higher than in the general population. All statistical analyses were performed in IBM SPSS statistics 23. In conclusion, although maternal and perinatal outcomes in pregnant WLHIV have improved over the past two decades, a high rate of adverse perinatal outcomes related to placental dysfunction (SGA, preeclampsia) persist. Our findings highlight the need for optimized prenatal care and further research to develop targeted interventions for WLHIV.

## 1. Introduction

Each year, an estimated 1.3 million women living with HIV (WLHIV) become pregnant [1]. Since 2016, the World Health Organization (WHO) has recommended that all people living with HIV should initiate lifelong antiretroviral therapy, including pregnant women [2]. This resulted in a marked increase in the proportion of pregnant WLHIV under treatment coverage at the time of conception, from 47% [38–55%] in 2015 to 84% [72 to >98%] in 2024 [1].

The expanded treatment access has subsequently improved maternal health outcomes and has led to a substantial reduction in vertical transmission. The baseline risk of transmission without intervention (25% to 30%) is now <0.5% to 2% in high-income countries with contemporary antepartum, intrapartum, and postnatal interventions [3,4,5,6]. Untreated maternal HIV infection has been consistently associated with elevated risks of adverse perinatal outcomes [7,8], including preterm birth, low birth weight (LBW), small-for-gestational-age infants (SGA), and stillbirth. The risk of adverse perinatal outcomes increased with more advanced HIV disease. While antiretroviral treatment (ART) has demonstrated significant benefits in reducing maternal morbidity and mortality while preventing vertical HIV transmission, its precise impact on improving perinatal outcomes remains controversial. While some studies report declining rates of preterm birth, low birth weight, and stillbirths with expanded ART use [9,10,11], others demonstrate no significant improvement in these outcomes for ART-treated WLHIV compared to HIV-negative controls [12,13,14,15,16,17,18,19]. Furthermore, ART regimens recommended during pregnancy have evolved over the years. The impact of these changing regimens on perinatal outcomes remains a topic of ongoing debate, with conflicting findings reported in the literature [20,21,22,23,24,25].

Current evidence on HIV, ART and perinatal outcomes has significant limitations. Most studies focus on African populations, where elevated rates of adverse perinatal outcomes—including preterm birth, LBW and SGA babies, hypertensive disorders of pregnancy and stillbirth—may be potentially affected by other demographic, behavioral, nutritional or clinical risk factors [26]. Comprehensive information regarding antenatal assessment, fetal ultrasonographic follow-up or virological parameters is frequently lacking. Furthermore, while accurate pregnancy dating is essential for defining adverse perinatal outcomes, existing studies show inconsistent gestational dating quality, with a subsequent potential for bias in perinatal data.

This study aims to analyze a well-characterized cohort of WLHIV at a tertiary referral center in a high-income country (Hospital Clínic of Barcelona, Barcelona, Spain) across two decades (2000–2010 vs. 2011–2021). We compare demographic characteristics, virological parameters, and treatment regimens between periods and assess how these changes over time may have influenced maternal and perinatal outcomes.

## 2. Materials and Methods

We conducted a retrospective cohort study of WLHIV who delivered at a tertiary referral center (Hospital Clínic de Barcelona, Barcelona, Spain) between January 2000 and December 2021. Cases involving delivery prior to 23 weeks of gestation were excluded from the study. Eligible patients were identified using the electronic hospital records database. Data were extracted from electronic records, anonymized and stored in password-protected electronic files in a specific database prospectively over the years. The data collected included items on maternal demographics, social and medical history, obstetric history, HIV laboratory investigations, ART, antenatal care, delivery, maternal and neonatal outcomes. Low educational level was defined as having either no formal education or only primary education completed. Undetectable viral load was considered if viral load was below 50 copies/mL. Gestational age was calculated based on the crown-rump length (CRL) in the first-trimester scan, except for those who booked late, who instead had dating scans in the second or third trimester (biparietal diameter used if BPD < 60 mm, or head circumference if BPD > 60 mm and uncertain last menstrual period) [27]. Preterm birth was defined as delivery before 37 + 0 weeks. Preeclampsia was defined as new-onset hypertension after 20 weeks of gestation (blood pressure ≥ 140/90 mmHg on two separate occasions at least 6 h apart) and proteinuria (protein/creatinine ratio ≥ 0.3 mg/mg or ≥300 mg/24 h urine collection) [28]. Birthweight centiles were calculated using normality curves validated in our population, adjusted by gestational age at birth and gender [29]. SGA was defined as birthweight < 10th centile, very small for gestational age (vSGA) was defined as birthweight < 3rd centile and large for gestational age (LGA) was defined as birthweight > 90th centile [30]. The study was registered and approved by the Hospital Clínic Ethic Committee (code HCB/2024/1232).

To compare trends in patient baseline characteristics, virological, obstetric and perinatal outcomes over time, cases were divided into two ten-year study periods according to the date of delivery: 2000–2010 (period 1) and 2011–2021 (period 2). For those WLHIV who had more than one pregnancy in the study period, each pregnancy was treated as a separate case.

Quantitative variables were assessed using Shapiro–Wilk’s test for normality, and normally distributed variables were compared using the *t*-test and expressed as mean and standard deviation (SD). Non-normally distributed quantitative variables were compared using U-Mann–Whitney test and expressed as median and interquartile range (IQR: p25–75). Qualitative variables were compared using Chi-square and Fisher’s exact test. To estimate the effect of the different sociodemographic, epidemiological and clinical-virological variables, logistic regression was used for binary dependent variables. All statistical analyses were performed in IBM SPSS Statistics 23. *p* < 0.05 was considered statistically significant.

## 3. Results

A total of 458 WLHIV were included (318 in period 1; 140 in period 2).

Baseline characteristics are detailed in Table 1. Maternal age at conception was similar between groups and a higher proportion of Black and Latina women was observed in period 2. Additionally, we noticed a decrease in the number of women with low educational level and in the rate of smoking and substance use in the later period. A significant increase in the average body mass index (BMI) was observed, rising from 22.65 ± 3.74 kg/m^2^ in the first period to 24.38 ± 4.78 kg/m^2^ in the second period (*p* = 0.000). While the proportion of women underweight (BMI < 18) remained similar in both periods (4% vs. 3.7%, *p* = 1.000), the proportion of women with overweight (BMI > 25) increased notably from 19.2% in the first period to 39.3% in the second period (*p* = 0.000).

Virological and immunological parameters are presented in Table 2. In both periods, HIV was diagnosed before conception in most cases (82%), with less than 2% of cases diagnosed at the time of delivery. Sexual transmission remained the predominant route of HIV acquisition in both periods. However, we observed a significant reduction in the number of pregnant women who acquired HIV through injection drug use (20.8% vs. 5.7%, *p* < 0.001) and a significant decrease in the prevalence of HCV coinfection between the two periods (29.2% vs. 10.7%, *p* < 0.001). Notably, in the second period, 8% of WLHIV had acquired the infection through perinatal transmission.

A reduction in the proportion of women with detectable viral load was observed during both the first trimester (45.8% vs. 25.6%, *p* < 0.001) and at delivery (20% vs. 8.7%, *p* < 0.002) in the second period. Furthermore, a significant improvement in the immunological status of WLHIV was observed between the two periods. The CD4 nadir increased significantly, from 238 cells/μL in the first period to 327 cells/μL in the second period (*p* < 0.001). CD4 counts during the first and third trimesters were also higher in the second period. Finally, a significant improvement in the CD4/CD8 ratio was noted in both the first and third trimesters during the second period.

ART characteristics are summarized in Table 3. A significantly higher proportion of WLHIV were already on ART at the time of conception in the second period (61.6% vs. 72.9%, *p* =0.025). Furthermore, the proportion of women receiving ART during pregnancy rose from 89% to 97% in the second period with a significant increase in the proportion of women receiving ART during the first trimester (55% vs. 75.7%, *p* < 0.001). Significant changes were observed in the ART regimens over time. The use of non-nucleoside reverse transcriptase inhibitor (NNRTI)-based ART significantly decreased in the second period (48.3% vs. 26.4%, *p* < 0.001). In contrast, the use of integrase strand transfer inhibitor (INSTI)-based ART markedly increased in the second period (0.7% vs. 39.2%, *p* < 0.001). No differences were observed in the use of protease inhibitor (PI)-based ART (51.6% vs. 52.9%, *p* = 0.839), although a shift towards newer PIs, first to lopinavir and thereafter to darunavir, was observed. In the nucleoside reverse transcriptase inhibitor (NRTI) group, we observed a significant shift towards reduced use of zidovudine and lamivudine, and an increased use of tenofovir, emtricitabine and abacavir.

Regarding perinatal outcomes (Table 4), a significant reduction in the rate of elective cesarean sections was observed in the second period (56.0% vs. 42.9%, *p* = 0.008), accompanied by an increase in the proportion of spontaneous vaginal deliveries (24.8% vs. 44.3%, *p* = 0.008). The use of intrapartum zidovudine also sharply declined in the second period (93.3% vs. 55.4%, *p* < 0.001). Despite these changes, vertical transmission rates remained <0.5% across both periods, with only one neonate testing positive over 20 years. This case occurred in the 2000–2010 period and was attributed to a late maternal seroconversion during pregnancy (negative HIV test in the first trimester, spontaneous vaginal delivery at 35 + 6 weeks of gestation, intrapartum HIV-positive testing and no possibility of cesarean section or intrapartum zidovudine due to rapid delivery). A significant reduction in the overall preterm birth rate was observed in the second period (22% vs. 11.4%, *p* = 0.020), driven primarily by a decline in iatrogenic preterm births, although spontaneous preterm birth also experienced a non-significant reduction. Subsequently, few neonatal intensive care unit (NICU) admissions (21.4% vs. 11.6%, *p* = 0.035) were observed and mean birth weight significantly increased in the second period. However, the overall rate of SGA neonates remained high and stable between periods (26.4% vs. 20%, *p* = 0.323). No significant differences were observed in preeclampsia rates (early-onset [<34 weeks] or late-onset [≥34 weeks]), stillbirths, or neonatal mortality.

In the analysis of the potential associations between virological and immunological parameters in WLHIV and perinatal outcomes, the only statistically significant finding was a higher prevalence of preeclampsia in cases with vertical transmission route (25%) compared to other infection routes (6.7%; adjusted OR 4.69, 95% CI [1.180–18.617], *p* = 0.028). Similarly, PWID had a higher incidence of preterm birth (41.9%) than those with other transmission routes (14.3%; adjusted OR 2.92, 95% CI [1.510–5.640], *p* = 0.001). No significant associations were observed between viral load (first trimester or delivery) or CD4 count (booking or delivery) and perinatal outcomes (preterm birth, SGA, or preeclampsia). Regarding ART and perinatal outcomes, WLHIV with >10 years of ART exposure at the time of delivery had higher preeclampsia rates (15.9%) than those with <10 years (6.3%; aOR 2.72, 95% CI 1.05–7.04, *p* = 0.039). Conversely, PI-based ART was associated with lower preeclampsia prevalence (4.6%) vs. non-PI regimens (10.3%; aOR 0.43, 95% CI 0.20–0.92, *p* = 0.030). No other ART regimens were associated with adverse pregnancy outcomes.

## 4. Discussion

Over the past two decades, sociodemographic and virologic parameters among pregnant WLHIV in our setting have significantly improved, leading to an increase in vaginal deliveries without intrapartum zidovudine, and near-negligible vertical transmission rates. Perinatal outcomes have also improved, with a decrease in rates of stillbirth, vSGA and especially, a significant reduction in preterm births. However, SGA and preeclampsia rates remain persistently elevated in this population.

The epidemiological profile of pregnant WLHIV in our setting has significantly evolved over the past two decades, marked by an increased representation of Black and Latina women and a sharp decline in toxic substance use and low educational attainment. This demographic shift aligns with recent epidemiological trends reported by other groups across Catalonia and Spain [31,32]. While sexual transmission remained the predominant HIV acquisition route in both periods, we observed a significant decline in cases attributable to injection drug use (PWID), and an increasing proportion of WLHIV with perinatally acquired infection in the latter period. This emerging subgroup represents individuals born to women with HIV who have now reached childbearing age and may require specific attention due to challenges related to treatment adherence, long-term ART exposure, and difficulties achieving viral suppression [33,34,35,36]. The long-term consequences of ART exposure, the use of suboptimal therapies during childhood, and the effects of chronic inflammation and immune activation in this subgroup remain largely unknown for both the mothers and their newborns. Notably, in our cohort, perinatal transmission was associated with almost 5-fold increased risk of preeclampsia and almost 3-fold increased risk of SGA, although this last one did not reach statistical significance in the adjusted analysis. Other groups [37,38] have also reported an increased risk of SGA neonates in this population. Further research is needed to elucidate the underlying mechanisms and the potential role of prolonged ART exposure and chronic inflammation on this apparent higher risk of placental insufficiency. Finally, we observed a trend toward increasing BMI over time in pregnant WLHIV, with a significantly higher proportion of overweight women (BMI > 25) in the second period. This trend may be partly attributed to lifestyle changes but also to the shift toward INSTI-based ART regimens, which have become the preferred option according to WHO guidelines [39]. Although INSTI-based regimens are highly effective for viral suppression, its association with excessive weight gain remains a concern [40]. Excessive gestational weight gain and obesity have been independently associated with adverse birth outcomes, including hypertensive disorders, gestational diabetes, and preterm birth, in both HIV-infected and uninfected women [41,42].

Simultaneously, over the past decade, WLHIV in our cohort have shown significant immunological improvement, reflecting optimized disease management and enhanced treatment outcomes. A growing proportion of women are now receiving ART preconceptionally or initiating treatment early in pregnancy. This has led to a higher rate of viral suppression at the time of the first antenatal assessment, as well as improved CD4 counts and CD4/CD8 ratios. ART coverage has increased over time -reaching 97% of WLHIV in the second period- with significant changes in the composition of regimens over time. These advances in maternal health, ART efficacy, and sustained viral suppression among WLHIV over the past decade have enabled a standardized obstetric approach, with clinical management determined primarily by obstetric indications rather than HIV status. Consistent with recent findings published by the Working Group of the National Cohort of Pregnant Women Living with HIV and Their Children in Spain [43], we observed a significant reduction in elective cesarean section rates and intrapartum zidovudine use, alongside an increase in the proportion of spontaneous vaginal deliveries. Importantly, these changes did not impact the vertical transmission rate, which remained below 0.5% in both periods, with only one neonate testing positive over 20 years due to a late maternal seroconversion during pregnancy.

Regarding perinatal outcomes in our cohort, a sharp reduction in the rate of preterm birth was observed in the second period. While there has been no measurable change in preterm birth rates over the last decade in the general population [44], rates among WLHIV have declined from 22% to 11.4% in the same period, approaching the incidence in the general population (9–11%) [44]. In the multivariable analysis, the only significant association was a 3-fold increased risk of preterm birth in PWID compared to other transmission routes. This likely reflects poorer maternal health in this subgroup and the adverse effects of substance use on pregnancy outcomes. The higher prevalence of HCV coinfection in this subcohort may also contribute to the elevated prematurity risk, as reported by other groups [45,46]. No associations were found with other virological or immunological parameters. Recent studies have demonstrated that PLWHIV with low CD4/CD8 ratios exhibit heightened inflammation and immunosenescence, despite achieving viral suppression with ART [45,46]. Conversely, higher CD4/CD8 ratios during ART suggest improved immune restoration and reduced chronic inflammation. However, there is limited evidence regarding the impact of CD4/CD8 ratio restoration in pregnancy and its potential association with perinatal outcomes. Floridia et al. [47] described in a cohort of 934 women that WLHIV who entered pregnancy with a CD4/CD8 ratio below 0.3 were more than twice as likely to experience preterm delivery and four times less likely to achieve viral suppression by the third trimester. To our knowledge, no other studies have been published to confirm this association. In our cohort, we found no statistically significant link between CD4/CD8 ratios and perinatal outcomes, possibly due to missing data and limited sample size, which may have reduced statistical power. Although previous studies from high-income countries [48,49,50,51] showed a higher incidence of preterm birth among women who received ART—particularly PI-based regimens—when compared with no antiretroviral therapy or monotherapy, we observed no significant association between ART regimens or timing of initiation and preterm birth in our cohort. However, our sample size may have limited the power to detect differences between subgroups.

In contrast, perinatal outcomes associated with placental malperfusion remained stable and elevated compared to the general population in both periods. The proportion of SGA infants was consistently twice as high as that in the general population (exceeding 20% in both periods), aligning with previous reports from our group [52] and others [19]. Similarly, preeclampsia rates showed no significant improvement between periods, remaining at approximately 7%—a figure markedly higher than the 2–3% reported in the general Spanish population [53]. Only a non-significant decrease in vSGA and stillbirths was observed, which may rather reflect a better obstetric monitoring considering a high-risk pregnancy management protocol. The etiopathogenesis of SGA infants and preeclampsia in WLHIV remains poorly understood. Current hypotheses suggest that placental dysfunction, chronic inflammation, and ART-related factors may play significant roles [49,54,55,56]. In the multivariable analysis, the only significant association in our cohort was a 2.7-fold increased risk of preeclampsia among women who had been on ART for more than 10 years at the time of delivery, compared to those on ART for less than 10 years. This suggests a deleterious cumulative effect of long-term ART on WLHIV. Consistent with this finding—and as noted previously—perinatally infected women in our cohort had an elevated risk of developing preeclampsia. Conversely, we observed a lower risk of preeclampsia among WLHIV treated with PIs compared to those on other regimens. No significant differences were found SGA infants or preeclampsia in relation to other antiretroviral regimens, timing of ART initiation, or virological/immunological parameters. Data on the association between ART and preeclampsia are conflicting in the literature. While a 2015 meta-analysis of 28 studies did not find any clear association between HIV and hypertensive disorders of pregnancy (HDP) [57], 2019 and 2023 systematic reviews reported an increased risk of HDP and greater cardiometabolic risk among pregnant WLHIV on ART compared to non-ART users, particularly with older protease inhibitors such as lopinavir–ritonavir and indinavir–ritonavir [58,59]. Nevertheless, the high risk of bias among included studies limits the strength of the conclusions.

We acknowledge that our study may have some limitations. Firstly, while data were collected prospectively, the evolving nature of the database structure resulted in incomplete variables for some parameters. Secondly, the sample size of WLHIV was significantly smaller in the second period, which could impact the statistical analysis. This disparity can be explained by the notable decline in the age-standardized incidence rate (ASIR) of HIV diagnosis over the past 30 years worldwide [60,61]. Despite this, we considered a 10-year timeframe to be appropriate for the analysis, as it aligns with significant changes in HIV management and facilitates robust statistical comparisons. We also acknowledge the lack of an HIV-negative control group. Nevertheless, published data about perinatal outcomes from the general population in our setting have been used to compare and discuss the results obtained in our study. Finally, the small proportion of untreated women in our study limited our ability to identify significant associations between antiretroviral treatment and perinatal outcomes, although it reflects current standard-of-care in pregnant WLHIV.

The major strengths of our study include its 20-year longitudinal design, which enabled period-stratified trend analysis, and prospective data collection that minimized recall and selection biases. The present study includes a large cohort of very well-characterized pregnant WLHIV. By integrating sociodemographic, virological, immunological, and perinatal outcomes, this work provides one of the most comprehensive assessments of pregnancy outcomes among WLHIV in high-income settings to date.

## 5. Conclusions

Over the past two decades, both sociodemographic and virologic parameters among pregnant WLHIV in our setting have significantly improved. This progress has led to a sharp decline in prematurity rates and an increase in spontaneous vaginal deliveries—without the need for intrapartum zidovudine—while maintaining near-negligible vertical transmission rates. However, these improvements have not translated into better perinatal outcomes related to placental dysfunction, as rates of small-for-gestational-age (SGA) infants and preeclampsia remain persistently elevated in this population.

Our findings highlight the need for optimized prenatal care, including close monitoring for placental insufficiency, as well as further research to identify the underlying mechanisms of adverse outcomes and develop targeted interventions for WLHIV.

## Figures and Tables

**Table 1:** Baseline Characteristics of WLHIV across two periods.

Characteristic	2000–2010 Period (n = 318)	2011–2021 Period (n = 140)	*p*-Value
**Maternal age (years);** mean ± SD	32.38 ± 5.37	32.88 ± 6.05	0.376
**Ethnic origin;** n (%)			
**Black**	33 (10.4)	28 (20)	**0.007**
**White**	246 (77.4)	67 (47.9)	**0.000**
**Asian**	0 (0.0)	1 (0.7)	0.306
**Latina**	22 (6.9)	36 (25.7)	**0.000**
**Other**	17 (5.3)	8 (5.7)	0.827
**Low educational level;** n (%) **(n = 451)**	134 (42.3)	32 (23.9)	**0.000**
**Smoking in pregnancy;** n (%)	134 (42.1)	37 (26.4)	**0.002**
**Substance use in pregnancy ^1^;** n (%)	53 (16.7)	13 (9.3)	**0.043**
**Nulliparity;** n (%)	140 (44.0)	51 (36.4)	0.150
**BMI (Kg/m^2^);** mean ± SD **(n = 435)**	22.65 ± 3.74	24.38 ± 4.78	**0.000**
**BMI < 18;** n (%)	12 (4%)	5 (3.7%)	1.000
**BMI > 25;** n (%)	58 (19.2%)	53 (39.3%)	**0.000**

BMI, body mass index. ^1^ Referring to substance other than tobacco.

**Table 2:** Comparison of virological and immunological parameters in WLHIV across two periods.

Characteristic	2000–2010 Period (n = 318)	2011–2021 Period (n = 140)	*p*-Value
**HIV diagnosis**; n (%)			
**Before conception**	262 (82.4)	116 (82.9)	1.000
**During pregnancy**	50 (15.7)	23 (16.4)	0.890
**Intrapartum**	6 (1.9)	1 (0.7)	0.681
**Transmission route**; n (%)			
**Unknown**	8 (2.5)	9 (6.4)	0.058
**Sexual**	240 (75.5)	110 (78.6)	0.550
**PWID**	66 (20.8)	8 (5.7)	**<0.001**
**Blood transfusion**	3 (0.9)	1 (0.7)	1.000
**Vertical transmission**	0 (0)	12 (8.6)	**<0.001**
**Coinfection HCV**; n (%)	93 (29.2)	15 (10.7)	**0.000**
**Months undetectable** **pre-pregnancy ^1^**; n (%)	44 (29)	55 (43)	0.077
**Detectable viral load**; n (%)			
1st trimester ^2^	119 (45.8)	31 (25.6)	**0.000**
At delivery ^3^	63 (20)	12 (8.7)	**0.002**
**CD4 nadir (cells/μL) ^4^**; mean ± SD	238 (155)	327 (206)	**<0.001**
**CD4 count (cells/μL);** mean ± SD			
**1st trimester ^5^**	486 (237)	547(215)	**0.020**
**3rd trimester ^6^**	541 (266)	604 (275)	**0.025**
**CD8 count (cells/μL);** mean ± SD			
**1st trimester ^7^**	805 (331)	689 (330)	**0.024**
**3rd trimester ^8^**	796 (346)	737 (301)	0.196
**CD4/CD8 count;** mean ± SD			
**1st trimester ^7^**	0.718(0.392)	1.003 (0.525)	**<0.001**
**3rd trimester ^8^**	0.761(0.404)	0.974 (0.552)	**0.003**

PWID, people who acquired HIV through injection drug use. Data available for: ^1^ n = 180; ^2^ n = 381; ^3^ n = 454; ^4^ n = 345; ^5^ n = 355; ^6^ n = 446; ^7^ n = 206; ^8^ n = 252.

**Table 3:** Antiretroviral therapy characteristics in WLHIV across two periods.

Characteristic	2000–2010 Period (n = 318)	2011–2021 Period (n = 140)	*p*-Value
**Months of ART** **pre-pregnancy ^1^;** mean ± SD	39.89 (51.92)	47.62 (68.27)	0.320
**Timing of ART;** n (%)			
Taking ART at conception	196 (61.6)	102 (72.9)	**0.025**
ART receiving in 1st trimester	175 (55)	106 (75.7)	**<0.001**
Anytime during pregnancy	284 (89.3)	136 (97.1)	**0.005**
ART regimen *; n (%)			
NRTIs	120 (99.2)	88 (100%)	1.000
NNRTI-based ART	73 (60.3)	31 (35.2)	**<0.001**
PI-based ART	57 (47.1)	39 (44.3)	0.779
INSTI-based ART	1 (0.8)	28 (31.8)	**<0.001**
Type of PI *; n (%)			
Lopinavir	14 (11.6)	20 (22.7)	**0.037**
Darunavir	0 (0)	11 (12.5)	**<0.001**
Ritonavir	31 (25.6)	17 (19.3)	0.320
Atazanavir	5 (4.1)	5 (5.7)	0.746
Indinavir	4 (3.3)	0 (0)	0.140
Saquinavir	17 (14.0)	3 (3.4)	**0.009**
Nelfinavir	15 (12.4)	0 (0)	**<0.001**
Type of NRTIs *; n (%)			
Zidovudine	83 (68.6)	20 (22.7)	**<0.001**
Abacavir	15 (12.4)	31 (35.2)	**<0.001**
Tenofovir	21 (17.4)	46 (52.3)	**<0.001**
Lamivudina	105 (86.8)	48 (56.5)	**<0.001**
Emtricitabine	7 (5.8)	42 (47.7)	**<0.001**

ART, antiretroviral treatment¸NRTIs, nucleoside reverse transcriptase inhibitor; NNRT, non-nucleoside reverse transcriptase inhibitor; PI, protease inhibitor; INSTI, integrase strand transfer inhibitor. ^1^ n = 344. * Sample sizes excluding WLHIV without treatment, n = 209.

**Table 4:** Comparison of perinatal outcomes among pregnant WLHIV across two periods.

Characteristic	2000–2010 Period (n = 318)	2011–2021 Period (n = 140)	*p*-Value	Adjusted *p*-Value ^a^
**Mode of delivery**; n (%)				
Elective C-section	178 (56.0)	60 (42.9)	**0.001**	**0.008**
Intrapartum C-section	49 (15.4)	15 (10.7)	0.192	0.910
Operative vaginal delivery	10 (3.1)	3 (2.1)	0.763	0.658
Spontaneous vaginal delivery	79 (24.8)	62(44.3)	**<0.001**	**0.008**
**Intrapartum zidovudine**; n (%)	294 (93.3)	77 (55.4)	**<0.001**	**<0.001**
**GA at delivery (weeks);** mean ± SD	37.5 ± 2.8	38.3 ± 2.4	**0.003**	**0.011**
**Preterm birth;** n (%)	70 (22)	16 (11.4)	**0.009**	**0.020**
Spontaneous	40 (12.6)	12 (8.6)	0.263	0.120
Iatrogenic	28 (8.8)	5 (3.6)	**0.050**	**0.037**
**Birth weight (g);** mean ± SD	2822.4 ± 682.0	3006.6 ± 650.5	**0.007**	0.155
**Birth weight centile;** mean ± SD	32.82 ± 1.84	31.13 ± 2.63	0.830	0.218
**Gestational age and gender adjusted birth weight;** n (%)				
SGA	84 (26.4)	28 (20)	0.158	0.323
vSGA	52 (16.4)	12 (8.6)	**0.028**	0.054
AGA	197 (61.9)	97 (69.3)	0.140	0.075
LGA	33 (10.4)	14 (10.4)	1.000	0.108
**Preeclampsia;** n (%)	23 (7.2)	10 (7.2)	1.000	0.636
Early (<34 weeks)	8 (2.5)	2 (1.4)	0.730	0.061
Late (>34 weeks)	15 (4.7)	8 (5.7)	0.647	0.801
**Stillbirth;** n (%)	6 (1.9)	1 (0.7)	0.681	0.359
**Neonatal death;** n (%)	1 (4.0)	0 (0)	0.180	0.995
**Adverse perinatal outcome;** n (%)				
Apgar 5′ < 7	8 (2.5)	3 (2.2)	1.000	0.565
pH UA < 7.20 ^1^	61 (20.3)	24 (21.4)	0.786	0.912
NICU admission	67 (21.4)	16 (11.6)	**0.012**	**0.035**
Neonate HIV positive ^2^	1 (0.4)	0 (0)	1.000	

GA, gestational age; SGA, small for gestational age; vSGA, very small for gestational age; AGA, adequate for gestational age; LGA, large for gestational age; pH UA, pH umbilical artery; NICU admission, neonatal unit admission. ^a^ *p*-value for comparison between 2000–2010 and 2011–2021 adjusted by: maternal age, smoking in pregnancy, substance use in pregnancy, ethnicity, BMI, nulliparity, low educational level, TAR before pregnancy or from first trimester of pregnancy, undetectable viral load and CD4 at delivery. Data available for: ^1^ n = 413; ^2^ n = 383.

## Data Availability

The raw data supporting the conclusions of this article will be made available by the authors on request.
